# Emotional Connotations of Musical Instrument Timbre in Comparison With Emotional Speech Prosody: Evidence From Acoustics and Event-Related Potentials

**DOI:** 10.3389/fpsyg.2018.00737

**Published:** 2018-05-15

**Authors:** Xiaoluan Liu, Yi Xu, Kai Alter, Jyrki Tuomainen

**Affiliations:** ^1^Department of Speech, Hearing and Phonetic Sciences, University College London, London, United Kingdom; ^2^Faculty of Linguistics, Philology and Phonetics, University of Oxford, Oxford, United Kingdom; ^3^Institute of Neuroscience, Newcastle University, Newcastle upon Tyne, United Kingdom

**Keywords:** musical timbre, emotional speech prosody, emotion, ERP, N400

## Abstract

Music and speech both communicate emotional meanings in addition to their domain-specific contents. But it is not clear whether and how the two kinds of emotional meanings are linked. The present study is focused on exploring the emotional connotations of musical timbre of isolated instrument sounds through the perspective of emotional speech prosody. The stimuli were isolated instrument sounds and emotional speech prosody categorized by listeners into anger, happiness and sadness, respectively. We first analyzed the timbral features of the stimuli, which showed that relations between the three emotions were relatively consistent in those features for speech and music. The results further echo the size-code hypothesis in which different sound timbre indicates different body size projections. Then we conducted an ERP experiment using a priming paradigm with isolated instrument sounds as primes and emotional speech prosody as targets. The results showed that emotionally incongruent instrument-speech pairs triggered a larger N400 response than emotionally congruent pairs. Taken together, this is the first study to provide evidence that the timbre of simple and isolated musical instrument sounds can convey emotion in a way similar to emotional speech prosody.

## Introduction

Music and speech are primary means for humans to communicate emotions (Buck, 1984). A considerable amount of studies have shown that affective music and speech are similar in many psychoacoustic dimensions, e.g., pitch, intensity and duration, which have been given extensive attention over a long period in terms of their cross-domain similarities (cf. Juslin and Laukka, 2003). Another important acoustic dimension is timbre, which is a multidimensional auditory property enabling listeners to distinguish between sounds that have equal pitch, loudness and duration (Giordano and McAdams, 2010). Timbre plays an important role in music construction (Menon et al., 2002), given the fact that composers rely on instrumentation (i.e., selection of different instruments) to depict the color and emotion of their music (Schutz et al., 2008). Despite its importance, timbre is not as well researched as pitch, intensity and duration (Holmes, 2011; Eerola et al., 2012). Only recently has timbre, especially musical timbre, attracted a reasonable amount of scholarly interest. In this study, we will further explore musical timbre, particularly in terms of the emotional connotations of musical timbre of isolated instrument sounds, with emotional speech prosody as a reference.

### The Relationship Between Musical Timbre and Emotion

Auditory processing of timbre requires perceptual integration of spectral and temporal dimensions (Griffiths and Warren, 2004). The significance of timbre in auditory processing is evidenced from the fact that even in infancy, humans can differentiate and memorize different types of timbre (Trehub et al., 1990); moreover, the processing of timbre in musical stimuli is more efficient compared to non-musical stimuli that are matched for complexity (Christmann et al., 2014), which shows that our brain is tuned for timbral differences in musical stimuli. In music theory, timbre is an effective platform for conveying composers’ underlying intentions and inducing emotions from listeners (Boulez, 1987; Gabrielsson, 2001). Early empirical evidence for the association of timbre with emotion can be found in Scherer and Oshinsky (1977) where systematic change in timbre (spectral filtering and envelope manipulation) led to listeners’ attribution to different emotions. Studies have also shown that timbre could be more immediate to the recognition of emotion than other music cues which usually take longer to process (cf. Eerola et al., 2012), as evidenced from the findings that listeners could distinguish emotion categories based on very short musical excerpts (e.g., Peretz et al., 1998; Bigand et al., 2005; Krumhansl, 2010).

Studies on polyphonic musical timbre (i.e., timbre of more than one instrument) (e.g., Alluri and Toiviainen, 2010) show that the arousal dimension of emotion is strongly correlated with the high-low frequency energy ratio of spectrums. Recently, an in-depth study of monophonic timbre (i.e., timbre of one instrument) (Eerola et al., 2012) investigated emotional connotations of isolated instrument sounds through a series of perception experiments. It was found that affective dimensions (e.g., arousal and valence) of the instrument sounds were mainly determined by spectral (high-low frequency ratio), temporal (attack slope, i.e., the slope of the period in which the sound changes in intensity before reaching its steady-state) and spectro-temporal (spectral flux, i.e., an acoustic feature measuring the speed of change of a signal’s power spectrum) parameters. Listeners’ consistent ratings of valence and energy arousal of the instrument sounds across experiments further indicate that timbre is a primary cue of conveying musical emotions. Neurophysiological research (e.g., ERP) on musical timbre (Goydke et al., 2004) show that the MMN (mismatch negativity) could be triggered by the same melody played with different emotions (happiness and sadness) or instruments (violin or flute) as standards or deviants, hence leading to the conclusion that the brain is sensitive to both emotional and timbral variations in music. A follow-up ERP study (Spreckelmeyer et al., 2013) further extended this line of research by incorporating more timbral variations of each emotion (happiness and sadness) as standards. The results showed MMN can still be elicited under pre-attentive condition, suggesting that in spite of the variance, the standards were still grouped together as a unified emotional entity (Spreckelmeyer et al., 2013). Brain imaging reports also showed that during the perception of musical timbre, evidence for cognitive processing of emotion was found in the P200 time window, as suggested by the additional anterior cingulate cortex (ACC) activities (Meyer et al., 2006).

### Why Further Research Is Needed

The studies reviewed above demonstrate a strong connection between musical timbre and emotion. Nevertheless, a problem common to almost all of these studies is that timbre was not tested as an independent acoustic cue free from variations in other acoustic cues such as pitch, duration and intensity, i.e., acoustic features other than timbre were not strictly controlled in those studies. The study by Eerola et al. (2012) (reviewed above) has a relatively stricter control, but special musical effects (e.g., vibrato, flutter) were not filtered out. It is known that effects like vibrato and flutter involve modulations in pitch and intensity (Olson, 2003). Therefore, it is not clear whether it was timbre alone or the combination of many acoustic features that contributed to the perceptual judgment of emotion reported in those studies. Hence, greater effort with a much more focused attention on timbre alone would be necessary to advance our understanding of the emotional connotations of musical timbre.

In addition, to our knowledge there is not enough research that directly compares emotional connotations of the timbre of isolated instrument sounds with human affective speech. We believe it is worth further exploring the relations between the two domains. This is because firstly the human voice, as a crucial platform for conveying speaker’s emotion and attitude (Banse and Scherer, 1996; Gobl and Ní Chasaide, 2003), has long been compared to musical instruments. String instruments such as the violin and the guitar are classic examples of the approximation of musical instruments to human vocal expressions (Askenfelt, 1991). Secondly, there is neurophysiological evidence showing that string instrument timbre and the human voice elicit similar ERP responses (Levy et al., 2003). But the voice stimuli used in that study were sung tones, and emotion was not included as a factor. Hence, it is still unknown whether musical instrument timbre could be comparable to human affective speech.

Relatively more direct comparisons between language and music timbre have used affective priming paradigm through the lens of N400 with visually presented words as primes or targets (Painter and Koelsch, 2011; Steinbeis and Koelsch, 2011; Goerlich et al., 2012). Affective priming refers to the phenomenon where the processing speed of an affective stimulus (e.g., the word “happy”) becomes faster when preceded by stimulus of the same affective category (e.g., the word “sunny”) than that of a different category (e.g., the word “boring”) (Klauer and Musch, 2003). In the field of music, Steinbeis and Koelsch (2011) showed that musical instrument timbre could communicate emotion to musically trained and untrained listeners. Short musical chords (800 ms) subjectively rated as pleasant or unpleasant were used as primes followed by visually presented words congruent/incongruent with the emotional valence of the chords. Words emotionally congruent with the chords (e.g., pleasant sounding chords followed by the word “beauty”) triggered smaller N400 amplitude than words emotionally incongruent with the chords (e.g., pleasant sounding chords followed by the word “anger”). In another study, Painter and Koelsch (2011) focused on the meaning of out-of-context music sounds while controlling for emotion. A larger N400 amplitude was triggered by semantically incongruent sound-word pairs than that of semantically congruent sound-word pairs. Nevertheless, in these studies, timbre was not independent of the variation in other acoustic features such as pitch, intensity or duration, and so it is not clear if it was timbre or the combination of acoustic features that contributed to the N400 effect. More importantly, the use of visual linguistic stimuli in these studies did not allow the exploration of the underlying link between the acoustic properties and emotional connotations. It has long been known that there are close acoustic connections between affective music and speech (Juslin and Laukka, 2003). What is still lacking, however, is a clear demonstration of the parallel in emotional meaning between the two domains.

### The Present Study

The aim of the present study is to explore the auditory properties that carry emotional meanings in both music and speech. We applied an affective priming paradigm in which both speech and music stimuli were auditorily presented. The particular focus is on whether a low-level acoustic dimension of isolated musical instrument sounds, i.e., timbre, is capable of communicating emotions in a way similar to human emotional speech prosody. For human speech, much of the emotional contents have been argued to be conveyed with a size code, whereby acoustic correlates of physical mass are used to project a large or small body size in order to influence the behavior of the listener (Ohala, 1984; Xu et al., 2013a). The expression of anger, for example, is to scare off the listener by projecting a large body size. This is because in a physical confrontation, having a large body size stands a better chance of defeating the opponent. The expression of happiness is to attract the listener by projecting a small body size to show non-threat or mimicry of infant voice (Xu et al., 2013a), as well as willingness to play (Panksepp, 2005). The code involves three acoustic dimensions: fundamental frequency (*F*_0_), formant dispersion (proximity of adjacent resonances of the vocal tract as a function of its length) and timbre (Morton, 1977; Ohala, 1984), of which the present study is concerned mainly with the last one, although fundamental frequency is also partially involved.

Due to physical laws, a rough timbre (e.g., lion roar) correlates to a large body size (Morton, 1977; Fitch et al., 2002). Hence it would convey anger in both musical sounds and speech. A pure-tone like timbre (e.g., bird chirps), in contrast, correlates with small body size (Morton, 1977). Hence it would convey happiness in both music and speech. With regard to sadness, two types need to be considered: grieving sadness and depressed sadness (Xu et al., 2013a), which are signaled by different body size projections (Xu et al., 2013a). Grieving sadness projects a large body size due to its demanding nature, while depressed sadness has relatively neutral size projection due to its lack of communicative intention (Xu et al., 2013a). Correspondingly, the timbre of grieving sadness is associated with higher spectral energy than that of depressed sadness (Scherer, 1979; Xu et al., 2013a). In addition, grieving sadness is also associated with relatively high, flat and slow changing fundamental frequency (Xu et al., 2013a), presumably to convey a sense of helpfulness.

It is therefore likely that musical instrumental timbre may work in a similar way as in speech to convey different emotions. Examples can be found in orchestral works (e.g., Tchaikovsky’s *The Nutcracker*; Prokofiev’s *Peter and the Wolf;* Blackford’s *The Great Animal Orchestra: Symphony for Orchestra and Wild Soundscapes*) where instruments with rough timbre are used to portray angry and large characters while instruments with tone-like timbre are used to portray happy and small characters, as well as instruments with mournful (grieving/wailing) timbre, were used to portray sad and large characters. So far, however, there is no direct empirical support for size projection as a common emotional code shared by both music and speech.

The present study, therefore, was designed to make a direct comparison between musical instrument timbre and speech in terms of emotional meaning representations in the auditory domain by using emotional speech prosody, rather than visually presented words as in previous studies. This was done through the affective priming paradigm in an ERP experiment focusing on the N400 effect, with speech and musical instrument stimuli categorized into three emotions (anger, happiness, and sadness). As demonstrated by previous studies (Bostanov and Kotchoubey, 2004; Pell, 2005; Paulmann and Pell, 2010), discrete emotional meanings (e.g., anger, happiness, and sadness) communicated through speech prosody can be automatically identified online around 300–400 ms after speech onset. Our hypothesis is therefore that emotionally incongruent instrument-speech pairs would elicit larger N400 than emotionally congruent instrument-speech pairs.

Both the music and speech stimuli were acoustically analyzed for their timbral features of the stimuli to assess whether they were consistent with the size-code hypothesis (i.e., the body-size projection theory), using multiple measurements as indicators. Four acoustic features (**Table 1**) were selected for measuring the timbre of the stimuli (cf. Eerola et al., 2012). They are all important features contributing to the emotional connotations of timbre (Eerola et al., 2012). The features were extracted from the speech and instrument stimuli using the MIR toolbox [cf. MIRtoolbox User’s Guide 1.6.1 by Lartillot (2014) for further details]. It is worth pointing out that timbre in music may correspond only partly with timbre in speech because in speech literature, timbre is still a relatively vague term that lacks a precise definition. Nevertheless, the acoustic features (e.g., attack slope, spectral centroid, etc.) selected in this study have been shown to be important parameters for examining affective speech timbre by several studies (e.g., Banse and Scherer, 1996; Goudbeek and Scherer, 2010; Xu et al., 2013b).

**Table 1:** Definitions of the four timbral features selected for this study [cf. Eerola et al. (2012) and Lartillot (2014)].

Attack slope	The slope of the period in which the sound changes in intensity before reaching its steady-state
Spectral centroid	Geometric center of the spectrum
Ratio of high-low frequency energy (HF/LF ratio)	The ratio between the amount of high spectral energy and low spectral energy
Spectral flux	A measure of the speed of change of a signal’s power spectrum

We expect that angry speech and musical instruments would be associated with a rough sound quality signaling a large body size, which will be indicated by higher attack slope, higher spectral centroid, and higher ratio of high/low frequency energy. Happiness would be associated with pure-tone like sound quality signaling a small body size, which compared to anger, will be indicated by lower attack slope, lower spectral centroid, and lower ratio of high/low frequency energy (Stevens, 1998; Eerola et al., 2012; Xu et al., 2013a,b). This is because the rougher the voice, the flatter the spectrum due to the abundance of high-frequency energy, which means higher spectral centroid, higher ratio of high/low frequency; while the more pure-tone like the voice, the less amount of high-frequency energy and hence lower spectral centroid (Stevens, 1998). The pattern of sadness will depend on the type of sadness: grieving or depressed or grieving sadness. Compared to depressed sadness which signals a neural body size, grieving sadness would be associated with a rough sound quality, and it would signal a large body size acoustically characterized by higher attack slope, higher spectral centroid, and higher ratio of high/low frequency energy. Depressed sadness would be associated with a low volume of high-frequency spectral energy, lower attack slope and low spectral centroid (Xu et al., 2013a). With regard to spectral flux, it is likely that happiness would have a higher value (i.e., more fluctuating) than anger due to its lower ratio of high-low frequency energy. Sadness would correspond to different spectral flux values depending on whether it is depressed or grieving sadness.

## Materials and Methods

This study was carried out in accordance with the recommendations of UCL Research Ethics Committee with written informed consent from all subjects. All subjects gave written informed consent in accordance with the Declaration of Helsinki. The protocol was approved by the UCL Research Ethics Committee.

### Participants

Sixteen native speakers of Mandarin Chinese without music training background participated in the ERP experiment (8 females, age *M* = 26, *SD* = 2.1). Data from one participant was discarded due to excessive muscle artifacts. The participants reported no hearing or speech impairments.

### Stimuli

The speech database includes a pre-recorded Mandarin sentence (*Cui luya nian shu qu le*, meaning *Cui luya* has gone to read a book) produced in three emotions (anger, happiness, and sadness) by eight native Mandarin speakers (4 females, age *M* = 25.3, *SD* = 2.1) (different from those of the ERP experiment), with each rendition of the sentence per emotion repeated three times. A following perception validation test was carried out using another 20 native speakers of Mandarin (different from the speakers for the sentence recording and ERP experiments) (10 females, age *M* = 28.3, *SD* = 5.2) without music training background. They were asked to rate the level of each emotion on each sentence on a 1–5 scale which indicated the intensity of the emotion (1 = very weak; 5 = very strong). The top 4 rated sentences of each emotion category were selected as the stimuli for the following ERP experiment. The mean score for all selected sentences in each emotion category was above 4.5. For each emotion, the speech items were from 2 females and 2 males (all different speakers).

The music database was from McGill University Master Samples (MUMS) (Opolko and Wapnick, 2006). This database includes sounds of almost all instruments (110 sounds altogether). Following Eerola et al. (2012), all the instrumental sounds were equal in pitch (D #4). For the purpose of this study, the sounds were also equalized in duration (1000 ms). Moreover, the loudness of all the sound samples was adjusted in a way to ensure a perception of equal-loudness (around 71 dB). In addition, sounds that had special effect (e.g., vibrato) were removed from the dataset. The purpose of this was to guarantee that other than timbre, all the rest of the acoustic features of the music stimuli remained perceptually the same. The same participants as those for the validation test of speech stimuli were asked to categorize each of the 110 instrument sounds into one of the following categories: anger, happiness, sadness, and neutral (i.e., no obvious emotion). Then each sound (except the sounds categorized as neutral) was rated on a 1–5 scale which indicated the intensity of the emotion (1 = very weak; 5 = very strong). The top four rated sounds of each emotion category (anger, happiness, and sadness) were selected as stimuli for the ERP experiment. The mean score for all selected sounds in each emotion category was above 4.3. Angry instruments selected were: cornet, alto shawm, crumhorn and saxophone. Happy instruments selected were: harpsichord, marimba, vibraphone and piano. Sad instruments selected were: violin, bassoon, flute and oboe.

### The ERP Priming Experiment

The ERP experiment was aimed to compare musical instrument timbre and emotional speech prosody with the priming paradigm (the N400 effect): musical instrumental sounds were used as primes and emotional speech prosody as targets, following a similar approach where words were targets and musical sounds were primes (Steinbeis and Koelsch, 2011). Moreover, in this study, we used an explicit priming paradigm, i.e., tasks that directly require participants to judge the relatedness between primes and targets, for the reason that the N400 effect could be either absent (Painter and Koelsch, 2011) or small (Frey et al., 2014) if an implicit priming paradigm (i.e., tasks unrelated to the judgment of the relatedness between primes and targets) was used. In this study, there were altogether 9 instrument-speech pairs (AA = angry instrument-angry speech; HA = happy instrument-angry speech; SA = sad instrument-angry speech; AH = angry instrument-happy speech; HH = happy instrument-happy speech; SH = sad instrument-happy speech; AS = angry instrument-sad speech; HS = happy instrument-sad speech; SS = sad instrument-sad speech). In each pair, each emotion had 4 representations (i.e., the top 4 rated instruments or speech in each emotion, as reported in section “Stimuli”). Each representation of each pair was presented 20 times. Altogether there were 9 (pairs) ^∗^ 4 (speech) ^∗^ 4 (instruments) ^∗^ 20 (times) = 2880 trials. They were grouped pseudorandomly. The Inter-Stimulus-Interval between the prime and target was 1000 ms. After hearing each instrument-speech pair, the participants had 1000 ms to judge whether the emotions conveyed by the instrument and speech were congruent or not by pressing a mouse button (left = yes, right = no). The participants were informed of the relevant emotion categories (anger, happiness, and sadness).

### EEG Recording and ERP Computation

The EEG was recorded using a Biosemi ActiveTwo system with 64 Ag-AgCI electrodes mounted on an elastic cap. The offsets at each electrode were kept between ±20 mV. To detect eye movement-related artifacts, bipolar horizontal and vertical EOGs (electro-oculograms) were recorded. The average of left and right mastoids was used as the off-line reference to all electrodes. Analysis software was EEGLAB v. 12.0.2.04b (Delorme and Makeig, 2004). The EEG epochs were time-locked to the stimulus onset, and baseline corrected (-200 to 0 ms). The data was filtered off-line by a band-pass filter of 0.1–30 Hz. Trials with EOG-artifacts were rejected offline using the artifact detection tools in ERPLAB v. 3.0.2.1 (Lopez-Calderon and Luck, 2014). The moving window peak-to-peak threshold tool (moving window width: 200 ms, voltage threshold: 100 μV, window step: 20 ms) and the step-like artifacts tool (moving window width: 400 ms, voltage threshold: 35 μV, window step: 10 ms) were used to reject trials with these artifacts. On average 19% of the data was rejected for anger, 16% was rejected for happiness and 18% was rejected for sadness. ERPs were averaged from the time window of 200 ms pre-stimulus onset to 800 ms post-stimulus onset. There were 6 regions of interest (ROIs): left anterior (FP1, AF7, AF3, F5, F3, FC5, FC3); mid anterior (FPZ, AFZ, F1, FZ, F2, FC1, FCZ, FC2); right anterior (FP2, AF4, AF8, F4, F6, FC4, FC6); left central-posterior (C5, C3, CP5, CP3, P5, P3,PO3); mid central-posterior (C1, CZ, C2, CP1, CPZ, CP2, P1, PZ, P2, POZ); right central-posterior (C4, C6, CP4, CP6, P4, P6, PO4). Trials in which participants’ judgment did not conform to the pre-determined emotion categories were not used for statistical analyses, following Steinbeis and Koelsch (2011).

### Statistical Analyses

A series of one-way repeated-measures ANOVA were performed on the comparisons between emotions (anger vs. happiness, happiness vs. sadness, and anger vs. sadness) for the timbral features of the speech and music stimuli. For the ERP priming data, we focused on the N400 component as discussed in Section “The ERP Priming Experiment.” We calculated the average amplitude within the N400 time window (350 ms to 500 ms), which was then entered into a three-way repeated measures ANOVA to investigate the effects of prime, target and regions of interest (ROIs). A series of one-way ANOVA were also conducted for *post hoc* analyses.

### General Procedure

The general procedure was that the speech and music sounds were first rated on a 1–5 scale (1 = very weak; 5 = very strong) in terms of the intensity of the emotion the sounds conveyed. The top four rated sounds of each emotion category (anger, happiness, and sadness) were selected as stimuli for the following ERP priming experiment. The whole ERP experiment lasted for 3 h. It was split into three sessions, each of which lasted for an hour with a 5-min break every 30 min. Prior to the EEG recording, the participants had 2 min to practice in order to familiarize themselves with the tasks for the experiment.

## Results

### Behavioral Results

**Table 2** summarizes relations between the three emotions (A = anger, H = happiness, S = sadness) with regard to the four timbral features of the selected speech and musical instrument stimuli, respectively. More specifically, relations between anger and happiness were the same across all four timbral features of speech and instruments: happy speech and instruments had higher values than angry speech and instruments (H > A) in terms of spectral flux; with regard to attack slope, spectral centroid, and high-low frequency energy ratio, angry speech and instruments had higher value than happy speech and instruments (A > H). Sad speech and instruments had the lowest spectral centroid, with sad musical instruments having a higher centroid value than sad speech. Nevertheless, the patterns of sadness were not consistent between speech and instruments with regard to attack slope, high-low frequency energy ratio and spectral flux. A series of one-way repeated-measures ANOVA were performed on the comparisons between emotions (anger vs. happiness, happiness vs. sadness, and anger vs. sadness) for the speech and music stimuli. The significant comparisons (*p* < 0.017, Bonferroni corrected) were indicated in bold in **Table 2**.

**Table 2:** Relations between the three emotions (A, anger, H, happiness, S, sadness) with regard to the four timbral features of speech and musical instruments, respectively [significant comparisons (*p* < 0.017, Bonferroni corrected) are indicated in bold in the second line of each stimulus type].

	Attack slope	Centroid	HF/LF ratio	Spectral flux
Speech	A > H > S	A > H > S	A > H > S	H > A > S
	(**A > H**, **A > S**, H > S)	(A > H, **A > S**, **H > S**)	(A > H, **A > S**, H > S)	(H > A, **H > S**, **A > S**)
Mean values	A: 33.13	A: 4259.18	A: 1.13	A: 115.8
	H: 13.68	H: 3900.3	H: 0.97	H: 117.3
	S: 11.92	S: 1832.4	S: 0.56	S: 15.78
Musical instruments	A > S > H	A > H > S	A > S > H	H > S > A
	(**A > S**, **A > H**, S > H)	(**A > H**, **A > S**, H > S)	(**A > S**, **A > H**, S > H)	(H > S, H > A, S > A)
Mean values	A: 59.98	A: 3719.68	A: 3.06	A: 9.3
	H: 18.48	H: 2364.71	H: 0.52	H: 13.6
	S: 24.84	S: 2147.1	S: 1.11	S: 13.2

**Table 3** shows the mean response disagreement rate (compared with pre-determined emotion categories) and 95% CI for all the instrument-speech pairs. A two-way (prime and target) repeated measures ANOVA showed that the effects of prime, target and their interaction were significant: prime [*F*_(2,28)_ = 292.2, *p* < 0.001, $\eta_{p}^{2}$ = 0.95]; target [*F*_(2,28)_ = 5.93, *p* < 0.01, $\eta_{p}^{2}$ = 0.3]; interaction [*F*_(4,56)_ = 8.46, *p* < 0.001, $\eta_{p}^{2}$ = 0.38]. More specifically, when angry instruments were primes, the disagreement rate was lower (*M* = 3.3); when sad instruments were primes, the disagreement rate was higher (*M* = 8.03). This suggests that the listeners’ judgment accuracy on the congruence/incongruence of the target depends on the prime. No reaction time data was collected because the task was a delayed response task.

**Table 3:** The mean disagreement rate and 95% CI for each instrument-speech pair (AA, angry instrument-angry speech; AH, angry instrument-happy speech; AS, angry instrument-sad speech; HA, happy instrument-angry speech; HH, happy instrument-happy speech; HS, happy instrument-sad speech; SA, sad instrument-angry speech; SH, sad instrument-happy speech; SS, sad instrument-sad speech).

	AA	AH	AS	HA	HH	HS	SA	SH	SS
Mean disagreement rate	3.09	3.86	2.87	5.63	4.08	3.76	8.16	7.73	8.09
95% CI	[2.73, 3.46]	[3.51, 4.21]	[2.26, 3.47]	[5.11, 6.16]	[3.58, 4.58]	[3.41, 4.11]	[7.51, 8.81]	[7.24, 8.31]	[7.44, 8.73]

### N400 Results

**FIGURE 1:**
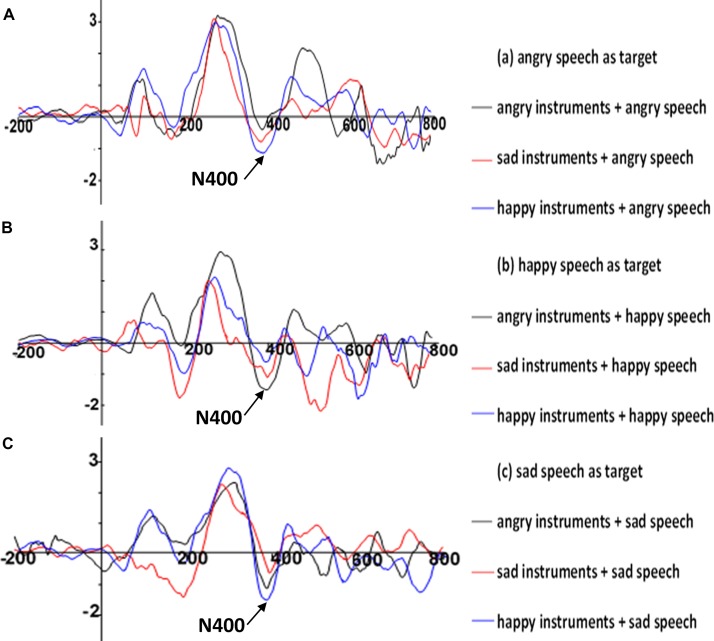
The N400 effect at Cz when angry speech **(A)**, happy speech **(B)**, and sad speech **(C)** was the target primed by musical instruments of different emotional categories. The average amplitude within the N400 time window was calculated.

**FIGURE 2:**
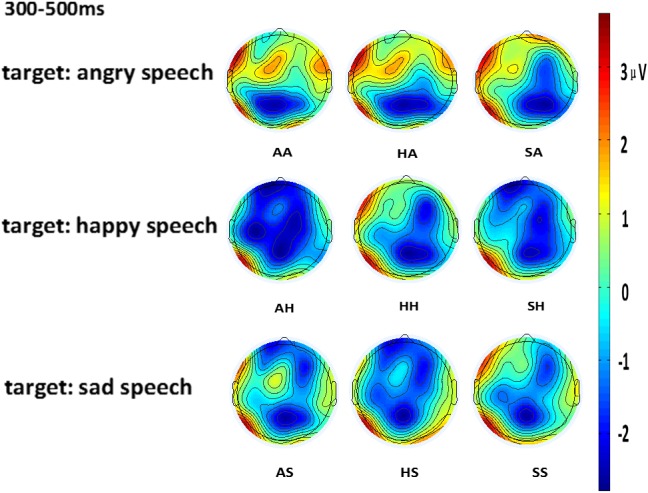
Scalp topography of the N400 effect for the nine instrument-speech pairs (AA, angry instrument-angry speech: AH, angry instrument-happy speech; AS, angry instrument-sad speech; HA, happy instrument-angry speech; HH, happy instrument-happy speech; HS, happy instrument-sad speech; SA, sad instrument-angry speech; SH, sad instrument-happy speech; SS, sad instrument-sad speech). The average amplitude within the N400 time window was calculated.

The selection of N400 time window in this study was from 350 to 500 ms based on visual inspection and previous literature on music and language priming (Painter and Koelsch, 2011). The average amplitude within the N400 time window was calculated. The N400 appeared larger in amplitude for emotionally incongruous instrument-speech pairs than the congruous pairs (see **Figure 1** for the ERP waveforms, **Figure 2** for the scalp topography, **Table 4** for the mean amplitudes and **Table 5** for the significance of the pairwise comparisons). Cz was selected for ERP presentation because this electrode site shows the strongest pattern for the incongruous vs. congruous differences between the nine instrument-speech pairs.

**Table 4:** The average amplitudes within the N400 time window (mean amplitudes) and 95% CI of the N400 for the nine instrument-speech pairs (AA, angry instrument-angry speech; AH, angry instrument-happy speech; AS, angry instrument-sad speech; HA, happy instrument-angry speech; HH, happy instrument-happy speech; HS, happy instrument-sad speech; SA, sad instrument-angry speech; SH, sad instrument-happy speech; SS, sad instrument-sad speech).

	AA	HA	SA	HH	AH	SH	SS	AS	HS
Mean amplitudes (μV)	-0.13	-0.33	-0.69	-0.90	-1.66	-1.01	-0.51	-0.83	-1.11
95% CI	[-0.2, -0.06]	[-0.41, -0.25]	[-0.74, -0.64]	[-0.95, -0.86]	[-1.71, -1.62]	[-1.05, -0.96]	[-0.55, -0.47]	[-0.87, -0.79]	[-1.15, -1.07]

**Table 5:** Significance (Bonferroni corrected) of the pairwise comparisons between the nine instrument-speech pairs (AA, angry instrument-angry speech; AH, angry instrument-happy speech; AS, angry instrument-sad speech; HA, happy instrument-angry speech; HH, happy instrument-happy speech; HS, happy instrument-sad speech; SA, sad instrument-angry speech; SH, sad instrument-happy speech; SS, sad instrument-sad speech).

	AA vs. HA	AA vs. SA	HA vs. SA	HH vs. AH	HH vs. SH	AH vs. SH	SS vs. AS	SS vs. HS	AS vs. HS
*p*	0.001	<0.001	<0.001	<0.001	0.001	<0.001	<0.001	<0.001	<0.001
*F*(df = 1, 14)	17.26	171.13	82.05	658.91	17.94	495.58	121.21	455.77	156.58
$\eta_{p}^{2}$	0.55	0.92	0.85	0.98	0.56	0.97	0.9	0.97	0.92

A three-way (prime, target, ROI) repeated measures ANOVA was carried out on the mean amplitude of N400 between 350 and 500 ms. The effects of prime, target and ROI were significant: prime [*F*_(2,28)_ = 28.67, *p* < 0.001, $\eta_{p}^{2}$ = 0.67]; target [*F*_(2,28)_ = 739, *p* < 0.001, $\eta_{p}^{2}$ = 0.98]; ROI [*F*_(5,70)_ = 1284.85, *p* < 0.001, $\eta_{p}^{2}$ = 0.99]. The interaction between the prime and target was significant [*F*_(4,56)_ = 275.74, *p* < 0.001, $\eta_{p}^{2}$ = 0.95]. Specifically, a follow-up one-way ANOVA (two levels: congruence; incongruence) showed a significant main effect of congruency [*F*_(1,14)_ = 459.27, *p* < 0.001, $\eta_{p}^{2}$ = 0.97], with incongruous prime-target pairs (*M* = -0.938, *SD* = 0.07) having significantly larger N400 amplitude than congruous prime-target pairs (*M* = -0.514, *SD* = 0.03). Moreover, within the incongruous prime-target pairs, a series of follow-up one-way repeated measures ANOVA (with Bonferroni corrected significance levels) showed that when anger was the target, sad primes (*M* = -0.69, *SD* = 0.09) had significantly larger amplitude than happy primes (*M* = -0.33, *SD* = 0.14) [*F*_(1,14)_ = 82.05, *p* < 0.001, $\eta_{p}^{2}$ = 0.85]; when happiness was the target, angry primes (*M* = -1.66, *SD* = 0.08) had significantly larger amplitude than sad primes (*M* = -1.01, *SD* = 0.08) [*F*_(1,14)_ = 495.58, *p* < 0.001, $\eta_{p}^{2}$ = 0.97]; when sadness was the target, happy primes (*M* = -1.11, *SD* = 0.07) had significantly larger amplitude than angry primes (*M* = -0.83, *SD* = 0.07) [*F*_(1,14)_ = 156.58, *p* < 0.001, $\eta_{p}^{2}$ = 0.92]. The results suggest that the modulation of the N400 amplitude of the target depended on the prime.

The interaction between targets and ROIs was also significant [*F*_(10,140)_ = 326.2, *p* < 0.001, $\eta_{p}^{2}$ = 0.96]. More specifically, when anger was the target, the mid central-posterior had the largest amplitude (*M* = -1.89, *SD* = 0.05) while the right-anterior had the smallest amplitude (*M* = 0.3, *SD* = 0.05); when happiness was the target, the mid central-posterior had the largest amplitude (*M* = -1.59, *SD* = 0.03) while the left anterior had the smallest amplitude (*M* = -0.98, *SD* = 0.03); when sadness was the target, the mid central-posterior had the largest amplitude (*M* = -1.9, *SD* = 0.03) while the right-anterior had the smallest amplitude (*M* = -0.15, *SD* = 0.01). These results suggest that the N400 effect was maximal in the mid central-posterior area across the three target emotions, while the smallest amplitude elicited depended on different target emotions.

## Discussion

### Timbral Features of Musical Instruments and Emotional Speech

In this study, we aimed to explore emotional connotations of musical timbre of isolated instrument sounds through the perspective of emotional speech prosody. Although the stimulus size is not big enough to allow for a robust generalization, the patterns demonstrated in **Table 2** could be seen as a qualitative trend underlying the stimuli: for each of the four timbral features, the relations between emotions were relatively consistent for speech and instruments, especially with regard to anger and happiness. In particular, angry instruments and angry speech were characterized by acoustic features that indicate a rough sound quality (e.g., higher attack slope and spectral centroid, higher ratio of high/low frequency energy, and lower spectral flux than those of happiness) while happy instruments and happy speech were characterized by features suggesting a pure-tone like sound quality (e.g., lower attack slope and spectral centroid, lower ratio of high/low frequency energy, and higher spectral flux than those of anger). Compared to happiness, anger has a higher attack slope which means a faster rise to its peak intensity, suggesting that the full volume of angry sounds is reached within a shorter time than that of happy sounds. These patterns are consistent with the predictions of body-size projection theory on emotion (Ohala, 1984; Xu et al., 2013a): anger corresponds to rough sound quality and large body-size projection aimed to scare off the listener; happiness corresponds to pure-tone like sound quality and small body-size projection aimed to show absence of threat and mimicry of infant voice. This further suggests that musical instruments could imply bio-behavioral meanings similar to those of affective speech, as discussed in Section “The Present Study.”

With regard to sadness, there are two kinds of sadness: grieving sadness and depressed sadness (Scherer, 1979). In this study, sad speech was produced more like depressed sadness while sad instruments rated by the listeners sounded more like grieving sadness (i.e., with more energy as indicated by the high-low frequency energy ratio). Depressed sadness is usually characterized by low amount of high spectral energy which means lower high–low frequency energy and spectral centroid as reflected in sad speech in this study; while the opposite is true for grieving sadness (Scherer, 1979; Xu et al., 2013a) as reflected in sad musical instruments in this study. Correspondingly, the lower value of attack slope of sad speech (*M* = 11.92) means a slower rise to the peak intensity of the sad speech, suggesting that sad speech reaches its full volume of intensity in a longer duration than sad musical instruments which has a higher value of attack slope (*M* = 24.84). These patterns are consistent with depressed and grieving sadness, respectively, which indicate two different body size projections: grieving sadness projects a large body size due to its demanding nature, while depressed sadness has relatively neutral size projection due to its lack of communicative intention (Xu et al., 2013a). Such correlation between size projection and sadness can be found in orchestral works such as *The Great Animal Orchestra: Symphony for Orchestra and Wild Soundscapes* (by Richard Blackford) where sad characters with a large body size were portrayed by instruments with mournful (grieving/wailing) timbre. Note that this by no means suggests that sad music is only associated with grief (since music can also convey depressed sadness). It was just that in this study instruments categorized into sadness had timbral features resembling grieving sadness. Future studies are needed to explore this further.

### N400

The results of the ERP experiment showed a clear N400 effect elicited by incongruence between musical instrument sounds and emotional speech prosody: emotionally incongruous instrument-speech pairs elicited larger N400 amplitude than emotionally congruous pairs, which confirms our hypothesis. The finding is in the same direction as that of Painter and Koelsch (2011) and Steinbeis and Koelsch (2011) where musical instrument sounds were shown (via priming) to communicate emotional meanings to musically trained and untrained listeners. Nevertheless, the music stimuli in the aforementioned studies did not have a strict control for other acoustic features such as fundamental frequency, intensity or duration. This means it could be the cohort of all acoustic features (not just timbre) that contributed to the N400 effect. The present study, in contrast, strictly controls all acoustic features except timbre, thus demonstrating a clearer picture of the emotional connotations of musical instrument timbre through the perspective of emotional speech prosody. In addition, this study further extends previous affective priming research on emotional meanings of music by showing that when targets were auditorily presented emotional speech prosody (rather than visually presented words as in previous studies), the N400 could still be elicited due to the emotional incongruence between music and speech, thus supporting previous claims that the N400 effect can exist regardless of domain or modality differences (Cummings et al., 2006). Note this effect was elicited when the task was an explicit priming task, i.e., participants were asked to judge directly whether the primes and targets were congruous or not in terms of emotion. Using an implicit task that is irrelevant to the judgment of the relatedness between primes and targets could either render the N400 effect absent (Painter and Koelsch, 2011) or small (Frey et al., 2014).

The N400 effect observed in this study also echoes the music perception model proposed in Koelsch and Siebel (2005). In that model, musical meaning could be directly accessed from low-level features of music such as pitch, intensity, and timbre, without the necessity of analysis of the whole piece of music. Empirical evidence for the model has been found in Painter and Koelsch (2011) where simple, stand-alone musical sounds could convey semantic meaning in a way similar to words. The present study further extends this line of research by showing that low-level musical features such as timbre can also compare directly with auditorily presented emotional speech prosody (not just visually presented words as in previous research) in terms of communicating emotional meanings. Therefore, the present study not only reveals a significant connection between two auditory domains (music and speech), but also highlights the cross-domain interchangeability between music and speech in communication of emotion, which echoes the idea that music and speech could share a similar code in conveying emotion (Juslin and Laukka, 2003). Compared to previous studies on music vs. speech similarities (as reviewed in Juslin and Laukka, 2003), the novel finding of the present study is that a single acoustic dimension such as the timbre of isolated musical instrumental sounds is enough to convey emotion in a way similar to human emotional speech that involves variation of multiple acoustic dimensions (pitch, duration, intensity, and voice quality). Future research along this line could focus on other low-level features such as pitch and intensity in comparison with human emotional speech prosody. Research effort toward this direction could contribute to a better understanding of cross-modal integration in the human brain.

## Conclusion

This study is focused on the comparison between musical instrument timbre and emotional speech prosody. For the first time, the timbre of simple, isolated musical instrument sounds was directly compared with emotional speech prosody via an affective priming paradigm. Therefore, this study is the first to provide empirical evidence for previous assumption that stand-alone musical instrument timbre could be similar to speech in triggering meaningful emotional connotations. This further supports the notion that timbre is an important means for composers to construct the emotional landscape of music, just as humans use voice to communicate emotions. These results thus add to the growing evidence for the cross-modal similarities between music and speech, especially in terms of emotion communication (Juslin and Laukka, 2003).

## Author Contributions

XL, YX, KA, and JT designed the experiments. XL collected and analyzed the data. XL, YX, KA, and JT wrote the manuscript.

## Conflict of Interest Statement

The authors declare that the research was conducted in the absence of any commercial or financial relationships that could be construed as a potential conflict of interest.
